# Uneven modulation of the annexin 1 system in osteoblast-like cells by dexamethasone^☆^

**DOI:** 10.1016/j.bbrc.2006.12.224

**Published:** 2007-03-09

**Authors:** Rosa M. Giner, Lucia Mancini, Ahmad M. Kamal, Mauro Perretti

**Affiliations:** a Departmento de Farmacologia, Universitat de Valéncia, València, Spain; b William Harvey Research Institute, Barts and The London, London, UK

**Keywords:** FPRL-1, Saos-2, Annexin A1, Dexamethasone, FPR, Interleukin-6, mRNA

## Abstract

We tested whether glucocorticoids modulated osteoblast expression of the annexin 1 system, including the ligand and two G-coupled receptors termed formyl-peptide receptor (FPR) and FPR-like-1 (FPRL-1). In Saos-2 cells, rapid up-regulation of FPR mRNA upon cell incubation with dexamethasone (0.01–1 μM) was observed, with significant changes as early as 2 h and a more marked response at 24 h; annexin 1 and FPRL-1 mRNA changes were more subtle. At the protein level, dexamethasone provoked a rapid externalization of annexin 1 (maximal at 2 h) followed by delayed time-dependent changes in the cell cytosol. Saos-2 cell surface expression of FPR or FPRL-1 could not be detected, even when dexamethasone was added with the bone modelling cytokines interleukin-6 or interleukin-1. The uneven modulation of the annexin 1 system (mediator and its putative receptors) in osteoblasts might lead to a better understanding of how these complex biochemical pathways become operative in bone.

Annexins are a family of structurally related proteins that exhibit Ca^2+^-dependent binding to anionic phospholipids and have been associated with several distinct biological functions [1]. Amongst different other tissues, annexins are present in cartilage and matrix vesicles isolated from chondrocytes where they have been proposed to influence bone matrix mineralization [2–4]. However, few studies have investigated annexin expression in bone cells: annexin 1 (Anx-A1), annexin 2, and annexin 5 are expressed in the human osteosarcoma cell line MG-63 [2] and rat primary osteoblasts and, at least Anx-A1, might modulate bone ontogeny as demonstrated for the murine palate [5]. In addition, annexin 2 favours osteoclast formation from bone marrow precursors [6] and promote osteoclast resorptive activity [7].

Within specific cell sources, Anx-A1 is predominantly found split between a cytosol and a membrane pool but, upon cell activation, it can be secreted (e.g., neutrophil [8]). Recent studies have pinpointed putative membrane receptors for Anx-A1 in members of the formyl-peptide receptor (FPR) family: whereas Anx-A1-derived N-terminal peptides interact with human FPR, the full-length protein is directly associated with FPR-like-1 (FPRL-1) on the cell surface of activated neutrophils [9]. Though initially identified on myeloid cells, it is now clear that several other cells types, including microglia and epithelial cells, can express these receptors [10].

It is of interest that Anx-A1 was originally identified as a mediator responsible for certain anti-inflammatory actions displayed by glucocorticoids (GC), drugs widely used in chronic inflammatory conditions and cancer. However, long-term therapies with glucocorticoids are known to be associated with secondary osteoporosis *in vivo* [11]. GC have diverse and complex effects on bone, their action being explained with increased parathormone release (favouring bone resorption), decreased recruitment of osteoblasts from osteoprogenitor cells, accelerated apoptosis of osteoblasts and osteocytes, and inhibition of bone formation [12]. Some of these effects might be secondary to modulation of two major determinants in bone cell functions, osteoprotegerin (OPG) and receptor activator of NF-κB (RANK) ligand (RANKL). In fact, GCs inhibit OPG production and stimulate RANKL expression in several osteoblastic lineage cells [13].

In the present study, we investigated the role of the Anx-A1 system in osteoblasts. We report initial description for the presence of this protein and its putative receptors, and investigate the modulation by GC and other bone-active mediators.

## Material and methods

Unless otherwise specified, all reagents were purchased from Sigma–Aldrich (Poole, UK).

*Cell culture and messenger RNA detection*. The human osteosarcoma cell line Saos-2 (ATCC, USA) was cultured in McCoy medium with 10% FCS (Gibco, Paisley, UK), 100 U/ml penicillin, and 100 μg/ml streptomycin, in atmosphere of 5% CO_2_/95% air at 37 °C and passaged two times a week. For mRNA analyses, cells (2 × 10^6^ cells/well) were cultured in 6-well plates; when sub-confluent, medium was replaced with RPMI without FCS and stimuli added 24 h later. Plates were washed with PBS and total cellular RNA was extracted with a Rneasy™ Mini kit following the manufacturer instructions (Qiagen Ltd., UK); RNA was then reverse transcribed with random primers (RETROscript™, Ambion Inc. TX, USA) and cDNA amplified using puRE Taq Ready-To-Go™PCR Beads (Amersham Biosciences, UK) [primers used and PCR conditions are listed in Table 1]. The expected product sizes for the products are as follows: 1095 bp for FPR, 1055 bp for FPRL-1, 1021 bp for Anx-1A, and 363 bp for GAPDH. The amplified products were run on 2% agarose gel, and densitometry analysis was performed using Scion Image, NIH software, USA.

*SDS–PAGE and Western blotting*. After different treatments, supernatants were collected and concentrated in Centricon™ centrifugal filter devices (YM-10, Millipore Ltd., UK Limited) at 4000 rpm for 30 min at 4 °C and cells were washed with 1 mM EDTA to remove cell surface-bound protein. Cell pellets were homogenized by sonication in 50 mM Tris buffer rich in protease inhibitors (1 mM phenylmethylsulphonylfluoride, 1.5 mM pepstatin A, and 0.2 mM leupeptin, pH 7.4); protein concentrations in supernatants were determined by the Bradford assay, adjusted to 2 mg/ml, mixed 1:5 with loading buffer (Pierce, PerbioScience, UK) before boiling for 5 min. Samples (20 μg proteins per lane), molecular weight markers (Bio-Rad Laboratories Ltd., UK), and human recombinant Anx-1A (50 ng) were subjected to 10% sodium dodecyl sulphate–polyacrylamide gel electrophoresis and transferred onto nitrocellulose membranes. Specific monoclonal antibodies (mAb) were used: anti-human Anx-1A antibody 1B was a generous gift of Dr. J.L. Browning (Biogen Corp, Cambridge, MS, USA), anti-human FPR antibody clone 5F1 was purchased from BD Biosciences Pharmingen, Oxford, UK; anti-human FPRL-1 antibody clone 6C7-3-A, was a generous gift of Dr. Duncan Henderson (AstraZeneca, Loughborouhg, UK). Densitometry analysis was performed using Scion Image, NIH software, USA.

*Flow cytometry*. Protein expression on the cell surface was monitored by flow cytometry, incubating 5 × 10^5^ cells with 5 μg/ml of primary antibodies (see section above) for 1 h at 4 °C, prior to addition of a FITC-conjugated rabbit anti-mouse IgG (Serotec, Oxford, UK), and analysis by flow cytometry with a FACScan flow cytometer (FACScalibur, Becton–Dickinson).

*Statistical analysis*. Results are expressed as means ± SEM, and data were analysed for statistical significance by ANOVA followed by Dunnett post hoc analysis.

## Results and discussion

### Anx-A1, FPR, and FPRL-1 expression in Saos-2 cells: effect of dexamethasone

**Fig. 1:**
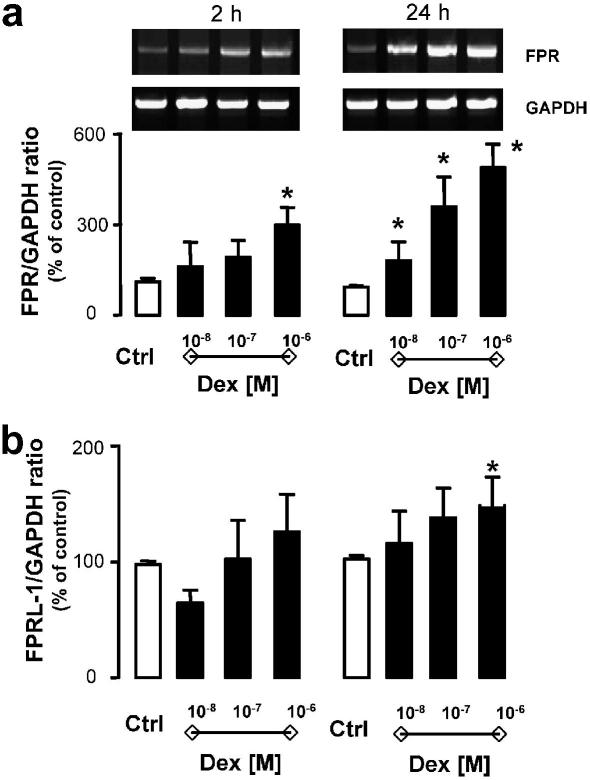
Effect of dexamethasone on Saos-2 FPR and FPRL-1 gene expression. Saos-2 cells were incubated for 2 and 24 h in the absence and presence of dexamethasone (0.01, 0.1, and 1 μM). Total RNA was then extracted and PCR performed for FPR (a) or FPRL-1 (b) gene expression. Autoradiogram of one representative experiment and densitometric analysis, normalized for corresponding GAPDH values, expressing the means of % referring to the control ± SEM of four independent experiments. *P* < 0.05, dexamethasone-treated vs. control cells.

The initial experiments focused on monitoring Anx-A1, FPR, and FPRL-1 expression in resting Saos-2 cells. At the message level, Anx-A1 was easily detected whereas either receptor type was scarcely detected. However, when dexamethasone (Dex) was added to Saos-2 cells, it produced rapid up-regulation of FPR mRNA (Fig. 1a). This was evident as early as 2 h post-addition, being more pronounced and fully concentration-related at the 24 h time-point (Fig. 1a). In contrast, Anx-A1 mRNA detection did not significantly differ in cells incubated with the steroid (not shown). FPRL-1 mRNA modulation was somehow intermediate, with lack of up-regulation at 2 h, but a significant increase seen at the 24 h time-point, though only for the highest concentration of Dex tested (Fig. 1b). This is at variance from what we have recently reported in monocytes and differentiated HL-60 cells, where a marked and significant effects of GC upon FPRL-1 mRNA and protein was evident within this time frame [14].

**Fig. 2:**
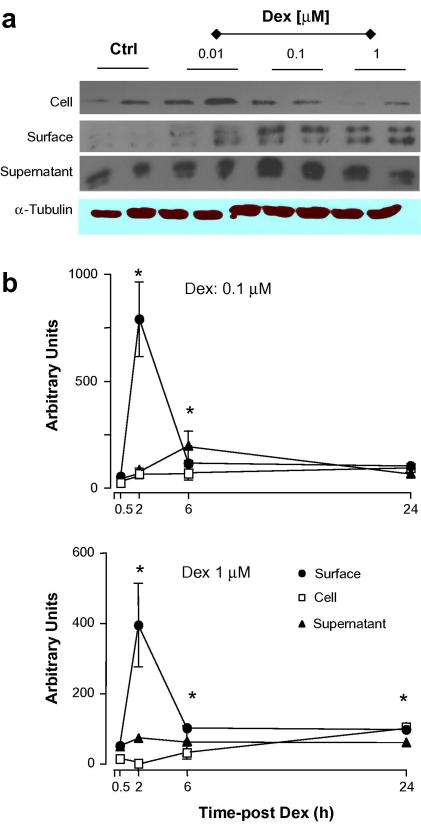
Kinetics of Anx-A1 protein expression induced by dexamethasone. Saos-2 cells were incubated with 1 μM dexamethasone (Dex) for the reported time-points, and Anx-A1 protein expression was determined in the medium, cell surface and cell lysate samples. (a) Shows representative blots, whereas (b) represents the densitometric analysis of three independent experiments, mean ± SEM of values shown as percent of control group (vehicle only). ^∗^*P* < 0.05, Dex-treated vs. respective control.

**Fig. 3:**
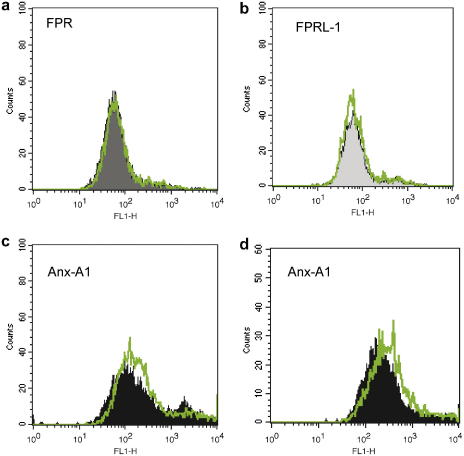
Dexamethasone and FPR, FPRL-1 or Anx-A1 protein expression in Saos-2 cells. Saos-2 cells were incubated with 0.1 μM dexamethasone for 48 h, and then stained with anti-FPR (a), anti-FPRL-1 (b) or anti-Anx-A1 (c,d) primary antibodies. In one case, panel d, cells were treated for 48 h with 0.1 μM dexamethasone + 1 ng/ml IL-6. Data are representative of three distinct experiments.

Next, we tested whether changes in transcription could be associated with alterations in protein levels. The results obtained were quite unexpected, since now Dex modified Anx-A1, but not FPR or FPRL-1, protein expression. Western blotting analysis indicated both a rapid and a delayed Dex-mediated up-regulation of Anx-A1 expression. In many cell types Anx-A1 exists as a cytosolic pool and a membrane-bound pool [15,16], and in vitro experimental conditions, the protein can also be recovered from the incubation medium, as in this case of activated neutrophils [8]. The significance of these pools is partially clear, and might be associated with distinct biological actions of the protein occurring both with non-genomic and genomic-derived pathways. For instance, a direct effect of Anx-A1 and other members of the family on gene transcriptional activity has been proposed [17] together with a rapid ability to affect cell function [18]. This apparent complexity is likely linked to the specific pool of Anx-A1 as well as to its post-transcriptional status: externalized Anx-A1 is thought to be highly serine phosphorylated [19,20]. Fig. 2a reports representative blots as obtained 2 h post-Dex: a clear concentration-dependent decrease of Anx-A1 from the cell surface is evident and associated with augmented levels in the cytosol (Fig. 2a). Cumulative data from multiple experiments with 0.1 and 1 μM Dex are in Fig. 2b: in both conditions, induction in the cytosol is concomitant with (or secondary to) a decrease of the cell surface pool and this is followed, temporally, by protein release in the medium. No detection for either FPR or FPRL-1 protein was obtained by Western blotting in resting Saos-2 cells or with any of the culture conditions just discussed (data not shown). This set of data was then validated by flow cytometry analysis. Dex had no effect on either FPR or FPRL-1 protein levels (Fig. 3). The validity of these partially negative data is genuine since the anti-FPR and anti-FPRL-1 monoclonal antibodies detected receptor expression on leukocyte samples run in parallel (data not shown), and published data confirm their effectiveness in flow cytometric assays [14,21,22].

Collectively, these sets of data indicate that Dex exerts exquisite and tightly controlled effects in Saos-2 cells, with up-regulation of Anx-A1 protein expression but not of its putative receptors. However, changes in mRNA were evident, especially for FPR, adding this receptor to the list of gene controlled by the steroid. Of interest, a recent study conducted with primary monocytes has reported fluticasone to markedly upregulate FPR expression and function [23]. Next we sought to determine if a second stimulus was required to achieve full up-regulation of FPR and FPRL-1 protein expression in the osteoblast.

### Anx-A1, FPR, and FPRL-1 expression in Saos-2 cells: effect of cytokine addition

**Fig. 4:**
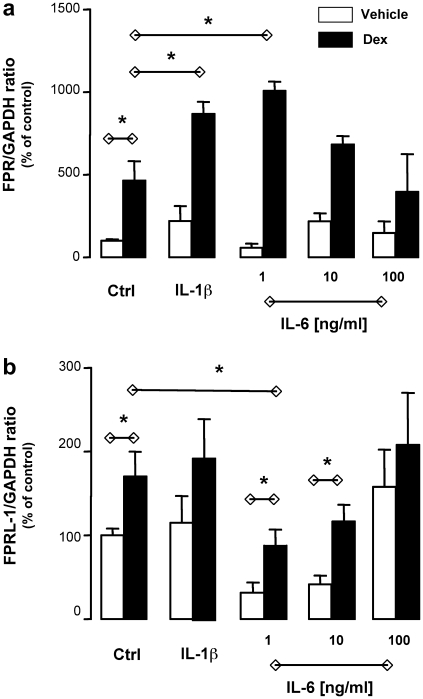
Synergistic effect of dexamethasone and IL-1β, or IL-6, on FPR mRNA expression. Saos-2 cells were incubated for 24 h in the absence and presence of dexamethasone (Dex; 0.1 μM) and IL-1β (10 ng/ml) or IL-6 (1, 10, and 100 ng/ml). Total RNA was then extracted and PCR performed for FPR (a) or FPRL-1 (b) gene expression. Data report the densitometric analysis of three independent experiments, mean ± SEM of values shown as percent of control group (vehicle only). *P* < 0.05, Dex-treated vs. control cells.

Interleukin (IL)-1 and IL-6 are potent cytokines known to affect bone metabolism. For instance, osteoblast-derived IL-6 was the major determinant of osteoclast function before discovery of the RANKL system; the same holds true for IL-1 [24]. In addition, in other compartments (e.g., liver) glucocorticoids and these two cytokines are known to operate in a synergistic/complementary fashion [25] and, for instance, in vitro glucocorticoids are required for optimal acute phase protein induction by IL-1 or IL-6 [26]. Saos-2 cell incubation with optimal concentrations of IL-1 and IL-6 did not significantly modify FPR mRNA expression; however, when cells were incubated also with Dex, a synergistic response was measured (Fig. 4a). Of interest, IL-6 effect disappeared by increasing the concentration indicative perhaps of multiple contrasting effects of the cytokine. In line with the data obtained with Dex alone, changes in FPRL-1 mRNA expression were more subtle and less marked (Fig. 4b). However, these changes in mRNA were not accompanied by changes in receptor expression on the cell surface, as investigated by flow cytometry: we tested several combinations of cytokines ± Dex, and different time-points, but were unable to unveil detection of the receptor (data not shown). On a similar note, Anx-A1 mRNA levels were not modified whereas subtle changes in protein expression were detected, though in line with what produced by Dex alone (see Fig. 3d for an example of Dex + IL-6). Altogether these experiments indicated that (i) Anx-A1 mRNA is quite stable in Saos-2 cells and (ii) FPR is most promptly modulated by these culture conditions. The biological significance of these findings will be addressed by separate studies, however the uneven modulation of the Anx-A1 system (mediator and its putative receptors) here revealed is quite a novel finding and may lead to a better understanding of how these complex biochemical pathways become operative in bone.

## Figures and Tables

**Table 1:** Specific primers and PCR cycling parameters for the proteins tested

Target	Specific Primer		PCR parameters			
	Forward	Reverse	Den	Ann	Elong	Cycles
FPR	GCGCAAGCTTACAGTCCAGGAGCAGACAAGATGGCGCAAGCTGT	GCGCGGCCGCTCACTTTGCCTGTAACTCCACCTCTGC	94 °C (1 min)	55 °C (1 min)	72 °C (1 min)	35
FPRL-1	TTCAGGTGCTGCTGGCAAGATGAAACCGCGGCGGATCCATGG	GCGCGGCCGCTCACATTGCCTGTAACTCAGTCTCTGC	94 °C (1 min)	65 °C (1 min)	72 °C (1 min)	35
ANXA1	CAATGGTATCAGAATTCCTCAAGGTGAAGGTG	GCGGCGTCGACTTAGTTTCCTCCACAAAGAGCCAC	94 °C (1 min)	55 °C (1 min)	72 °C (1 min)	27
GAPDH	GGAGTGAAC	GGCAGAGATGATGACCCTTTTGGC	94 °C (1 min)	60 °C (1 min)	72 °C (1 min)	30
